# Morphological adjustments enable sea urchins to sustain calcified structure function under ocean acidification

**DOI:** 10.1098/rspb.2025.1893

**Published:** 2025-11-26

**Authors:** Jonathan Y. S. Leung, Ivan Nagelkerken, Erin L. Pichler, Yujie Chen, Claire F. Jones, Zonghan Xie, Sean D. Connell

**Affiliations:** ^1^ Guangdong Provincial Key Laboratory of Marine Disaster Prediction and Prevention, Shantou University, Shantou, People’s Republic of China; ^2^ Southern Seas Ecology Laboratories, School of Biological Sciences, The University of Adelaide, Adelaide, South Australia, Australia; ^3^ School of Electrical and Mechanical Engineering, The University of Adelaide, Adelaide, South Australia, Australia

**Keywords:** adaptability, biomineralization, climate change, energy allocation, plasticity

## Abstract

Ocean acidification can reduce the size of calcified structures produced by marine calcifiers, raising questions about their competitiveness and persistence in future oceans. Yet, size reduction in calcified structures may represent a plastic response to ocean acidification if these structures remain functional. To test this hypothesis and examine whether morphological plasticity can influence the functionality of calcified structures, we assessed the effects of ocean acidification on the morphological, mechanical and chemical properties of the calcified structures of a sea urchin species prevailing at natural CO_2_ vents. We found that the rigid shells covering sea urchins’ bodies (‘tests’) were thinner and that they had smaller teeth and lower spine density at vents, but the mechanical performance of these calcified structures (mechanical resilience, wear resistance and bending strength) was maintained, possibly mediated by the capacity of sea urchins to sustain acid–base balance for calcification (i.e. increased Na/Ca). Our findings suggest that such morphological shifts in calcified structures may enable sea urchins to maintain structural performance under ocean acidification. Since ocean acidification is a slow process relative to the life cycle of sea urchins, some sea urchin species may acclimate, or even adapt, to ocean acidification so that their populations and ecological functions can persist in a future high-CO_2_ world.

## 1. Introduction

Ocean acidification driven by anthropogenic CO_2_ emissions has galvanized substantial research efforts over the past two decades because an early theory proposed that the reduced seawater carbonate saturation under ocean acidification can depress metabolism and stunt calcification in various marine calcifiers (e.g. coccolithophores, corals, bivalves and sea urchins), possibly causing their physiological dysfunction and even mortality [1]. In particular, negative effects of ocean acidification on calcification, such as reduced shell size and shell growth, have been widely reported in the literature [2,3], implying the sensitivity of calcifiers to ocean acidification (but see refs [4,5] for their mixed responses to ocean acidification).

While size reduction in calcified structures is generally regarded as a negative response to ocean acidification, this morphological change may represent a plastic response (i.e. a response of organisms to adjust to stressors), which enables organisms to maintain their normal performance and persist in the changing environment by altering their phenotype across organismal traits (i.e. phenotypic plasticity [6]). For instance, marine gastropods (*Cyclope neritea* and *Nassarius corniculus*) that can persist at natural CO_2_ seeps exhibit dwarfism (i.e. reduced shell size), which has a selective advantage in lowering the energy demand for building calcified structures [7]. The energy conserved can then be allocated to somatic maintenance and gonadal growth to maintain general health and reproductive output [8,9]. Likewise, marine polychaetes (*Platynereis dumerilii*) dwelling near natural CO_2_ vents have reduced body size but are able to persist locally by moderately downregulating metabolic rates as an adaptive response [10]. Thus, morphological shifts (or shape-shifting) in calcified structures may serve as a plastic response to environmental stress if these structures remain functional [11]. For example, despite the reduction in shell thickness, mussels (*Mytilus edulis*) can produce rounder, flatter shells under ocean acidification to enhance protection against predators [12]; corals (*Astroides calycularis*) colonized around natural CO_2_ vents can alter their skeletal phenotype, characterized by encrusting morphology with smaller size and fewer polyps but less porous and denser skeletons through genetic differentiation, which allows adaptation to the locally acidified environment [13]. While morphological plasticity may help calcifiers to accommodate ocean acidification, its potential benefits are rarely studied, especially in naturally acidified habitats.

The functional performance of calcified structures can also be affected by their chemical and structural properties at the microscale [14]. Ocean acidification has been shown to disrupt calcification, possibly weakening the integrity and mechanical strength of calcified structures. For example, ocean acidification can impair crystallographic control during calcification, leading to the production of porous, disordered structures ([15,16]; but see [17–19]). To overcome this challenge, calcifiers can maintain an optimal alkaline condition at the calcification site [20], depending on their capacity to regulate the acid–base balance of calcifying fluid. Calcifiers may also alter the organic content of calcified structures, which can influence their mechanical performance and chemical resistance to acidified seawater [16,21]. Furthermore, some calcifiers (e.g. echinoderms) can regulate the incorporation of magnesium ions (Mg^2+^) in calcified structures, which in turn alters their structural robustness [22]. Therefore, a comprehensive analysis of calcified structures, including structural, chemical and mechanical properties, is required to ascertain whether shape-shifting in calcified structures is a plastic response of calcifiers to ocean acidification.

In this study, sea urchins were chosen as the ideal candidate to assess whether morphological shifts can be induced by ocean acidification because sea urchins produce various types of calcified structures (i.e. teeth, tests and spines) with different functions. To obtain ecologically relevant results, ecological realism was taken into account by using sea urchins that persist in naturally acidified habitats (i.e. volcanic CO_2_ vents) within an entire generation. This field-based approach provides a higher interpretive value because extended time is often required for calcified structures to grow with discernible differences in morphology between treatments, which cannot be easily achieved by short-term laboratory experiments. The structural, chemical and mechanical properties of calcified structures were analysed to evaluate their integrity and functionality. Given that ocean acidification can hamper calcification in sea urchins [23], we hypothesized that if morphological shifts in calcified structures are a plastic response then this would result in a reduction in these structures’ size and/or weight in sea urchins at CO_2_ vents, but their integrity and functionality (e.g. mineral density and mechanical performance) would be maintained via acid–base regulation of calcifying fluid. By examining the morphology and functionality of calcified structures, this study may unlock a novel adaptive strategy that enables calcifiers to persist and even evolve in an acidifying ocean.

## 2. Material and methods

### (a) Study site and species

Volcanic CO_2_ vents are widely used as ‘natural laboratories’ for ocean acidification research and are typically characterized by areas of seawater naturally acidified by the CO_2_ emitted from vents. In this study, the natural CO_2_ vents at Whakaari/White Island, New Zealand, were selected as the study region, which constitutes a temperate rocky reef with the seabed covered by boulders. Benthic macroalgae (e.g. turf algae, coralline algae, fleshy algae, etc.) are abundant and widespread across the study region [24], while patches of kelp (*Ecklonia radiata*) are also found away from the influence of CO_2_ vents [25]. Sea urchin *Evechinus chloroticus* is the dominant herbivore found at both vent and nearby non-vent (i.e. control) sites. In this rocky reef, the CO_2_ plumes emitted from two separate shallow-water vents (6–10 m in depth) extend approximately 20 m and acidify the surrounding seawater. Relative to vents, we chose two random and interspersed control sites with ambient pH levels (pH approx. 8.10), which were located approximately 25 m from the vent sites at their respective locations (i.e., North and South; see ref. [25] for their locations). The vent sites had reduced pH levels (pH approx. 7.70–7.90) that approximately represent the end-of-century representative concentration pathways (RCP) 6.0–8.5 scenario (electronic supplementary material, table S1), whereas the pH of the control sites represents the contemporary pH levels of seawater. Using a pH logger (Sonde YSI 6600 V2), the natural diurnal variation of pH at the sites was approximately 0.15 units, while the seawater pH at the vent sites was consistently lower than the control sites by at least 0.2 units on average based on our multi-year observations (electronic supplementary material, table S2), suggesting that the vent sites are relatively stable over time [26]. For the vent sites, seawater samples were collected in February 2017 within the wider vent area (within approx. 20 m from the vents) and seawater pH was directly measured *in situ* for validation. While occasional drops in pH levels can be driven by vent activity that leads to spikes in CO_2_ release, these spikes occur on a short timescale (less than few hours) and cannot significantly influence the physiology and behaviour of organisms, which operate on a much longer timescale [27]. The temperature, nutrient and mineral concentrations of seawater were not significantly different between the vent and control sites (electronic supplementary material, table S1) [21,28]. Mercury was not detected in the seawater across sites (<0.005 mg l^−1^) and the level of detection limit is lower than seawater in the open ocean [29], meaning that the potential influence of mercury at our sampling region is negligible.

Adult sea urchins (*E. chloroticus*) were collected from four sampling areas in February 2017 based on the crossed combinations of seawater pH (ambient pH at controls and reduced pH at vents) and location (North and South). The sea urchins at the vent sites were collected within 1 m from the vents and had higher U/Ca ratios in their teeth than those at the control sites (electronic supplementary material, figure S1), indicating that their movement between control and vent sites is unlikely [30]. This is because adsorption of seawater uranium onto mineral and organic surfaces increases with decreasing pH [31], accounting for the widely observed inverse relationship between seawater carbonate concentration and U/Ca in calcified structures [32]. The higher uranium incorporation and thus U/Ca in the teeth substantiate that the sea urchins grew at the vent sites and had relatively small home ranges [33]. As the dispersal distance of sea urchin larvae before settlement, which can be up to hundreds of kilometres, is greater than the area of the vents, the differences observed between sea urchins at control and vent sites are probably owing to developmental plasticity rather than local adaptation. For the analyses of calcified structures, five healthy sea urchins of similar size (test height: 51.5 ± 6.6 mm) were selected from each sampling area to standardize the size for comparisons. This sample size can yield statistically robust results for the calcified structures of sea urchins based on the previous ocean acidification studies [34–37]. The test diameter and test height of sea urchins were measured to the nearest 0.1 mm with a calliper, whereas their body weight was measured to the nearest 0.1 g with an electronic balance. Then, the sea urchins were dissected to discard the fluid inside, followed by reweighing to obtain the total weight on a fresh-weight basis. The gut, gonad and Aristotle’s lantern were carefully separated and individually weighed. The weight of the test with spines was calculated by subtracting the total weight from the weight of gonad, gut and Aristotle’s lantern. The relative weight of each component (%) was calculated by dividing its wet weight by the total weight. Spine density was estimated by enumerating the spines on the interambulacral plate (approx. 4 cm^2^) of the test fragment. Spines were classified into three types based on their length: (i) <6 mm, (ii) 6–12 mm, (iii) >12 mm. Spines shorter than 6 mm are considered secondary spines, while those of 6 mm or longer are considered primary spines.

### (b) Morphological analyses of calcified structures

Three types of calcified structures, i.e. tests, spines and teeth, were analysed for each individual. These structures were cleaned, rinsed with Milli-Q water and then air-dried. To analyse the morphological and structural properties of the calcified structures, micro-computed tomography (micro-CT) was applied, which is a non-destructive imaging technique for producing high-resolution three-dimensional images. Micro-CT scanning was performed using a SkyScan 1276 system (Bruker, Kontich, Belgium), equipped with a 16-megapixel CMOS camera (4096 × 4096 pixels; maximum spatial resolution: 2.8 µm/pixel). The calcified structures were securely mounted onto the sample holder and then scanned in the scanning chamber. Since structural properties (e.g. mineral density or porosity) vary between different types of calcified structures, different acquisition settings were used to optimize the quality of CT-scan images (test—pixel size: 4 µm, voltage: 70 kV, current: 200 µA, exposure time: 820 ms, 0.25 mm Al filter; spine—pixel size: 3 µm, voltage: 60 kV, current: 200 µA, exposure time: 340 ms, no filter; tooth—pixel size: 5 µm, voltage: 90 kV, current: 200 µA, exposure time: 830 ms, 1 mm Al filter). For all types of calcified structures, a half rotation of 180° (rotation step: 0.2°) and frame averaging of 3 were used to reduce random noise. Image reconstruction was performed using the NRecon www.bruker.com software (Bruker, Kontich, Belgium) and the images were fine-tuned by applying smoothing (2), misalignment compensation (variable), beam hardening correction (50%) and ring artefact reduction (8).

The cross-sectional images of calcified structures were analysed using the software CTAn www.bruker.com (Bruker, Kontich, Belgium) to measure the size of samples at the microscale (e.g. test thickness, tooth length, spine length, etc.). Four ratios were calculated to assess whether the morphology of calcified structures was modified in response to ocean acidification: (i) test thickness/test diameter indicates the relative growth of tests in thickness; (ii) tooth length/test height indicates the relative size of teeth; (iii) tooth width/tooth length reflects the mechanical strength of teeth; (iv) spine width/spine length reflects the mechanical strength of spines. To analyse porosity, the threshold values of CT-scan images were set for each type of calcified structure in the binary threshold module, followed by using the plugins in the custom processing (thresholding, ROI shrink-wrap and three-dimensional analysis) to estimate the porosity of a calcified structure in a given volume (*n* = 5 samples per sampling area). The three-dimensional models of calcified structures showing the distribution of mineral density were visualized using the software CTVox www.bruker.com (Bruker, Kontich, Belgium; see electronic supplementary material, videos).

### (c) Mechanical testing of calcified structures

A previously described method was applied to prepare the samples for nanoindentation [21]. Briefly, the calcified structures were embedded in resin by cold mounting. The resin blocks were then polished to expose the cross section of the calcified structures using an automatic polishing machine (Struers TegraPol-11; Struers A/S, Denmark). To obtain a smooth surface, a series of three polishing steps were performed using MD Largo, MD Dac and finally MD Chem polishing discs with DiaPro Allegro/Largo 9 µm diamond suspension, DiaPro Dac 3 µm diamond suspension and OP-S 0.04 µm colloidal silica suspension, respectively. The hardness (*H*) and elastic modulus (*E*) of calcified structures were measured using a nanoindenter (IBIS, M/S Fischer-Cripps Laboratories, Australia) with a diamond Berkovich tip. Load-controlled indentation with a maximum load of 80 mN was used. Six random locations on the surface region of each calcified structure were indented to obtain an average value (*n* = 5 samples per sampling area). *H*/*E* ratio and *H*^3^/*E*^2^ ratio were calculated to represent the mechanical resilience of tests and wear resistance of teeth, respectively. The capacity of these calcified structures to deform plastically without fracture increases with the value of these ratios.

The bending strength of spines was assessed by three-point bending using a universal testing machine (Instron 5543, USA; load cell capacity ±50 N; Bluehill software), following a standard protocol [38]. Five spines (approx. 18 mm length) from each sea urchin were tested (*n* = 5 individuals per sampling area). The outer diameter of each spine, at the approximate midspan of the three-point bending test, was measured with a calliper prior to testing. Each spine was placed on the lower supports of a three-point bending apparatus, with a span of 12 mm. The actuator with upper pin positioned midspan was displaced downwards at a constant rate of 0.01 mm s^−1^. Load and displacement data were recorded at a sampling rate of 500 Hz. All the spines failed at the midspan. To estimate bending strength, which represents the stress that a spine can withstand before failure under a bending load, the spine was assumed to have a circular cross section of constant external diameter, and the following equation was applied:


$$\sigma_{b}=\frac{F_{max}L}{\pi r^{3}},$$

where *σ_b_* is the bending strength, *F*_max_ is the maximum load at fracture, *L* is the support span and *r* is the radius at the midspan of the tested spine.

### (d) Chemical analyses of calcified structures

The calcified structures were cleaned, rinsed with Milli-Q water, oven-dried, weighed and placed in an individual pre-weighed crucible (*n* = 5 samples per sampling area). The organic matter content of calcified structures was determined by weight loss upon ignition at 550°C in a muffle furnace for 6 hours.

Crystallinity, represented by relative amorphous calcium carbonate (ACC) content, was analysed by a Fourier transform infrared spectrometer (Spectrum 100, PerkinElmer, USA). The calcified structures were powdered and then transferred onto the sample holder of the spectrometer to obtain the infrared absorption spectrum, ranging from 650 to 1800 cm^–1^ with background correction (*n* = 5 samples per sampling area). The relative ACC content of calcified structures was estimated as the intensity ratio of the peak at 872 cm^–1^ to that at 713 cm^–1^ [17]. Crystallinity increases as the value of this ratio decreases.

The elemental concentrations of calcified structures were determined by laser ablation inductively coupled plasma mass spectrometry (LA-ICP-MS; RESOlution LR, Agilent 7900 x, Agilent Technologies, USA). Prior to analysis, the calcified structures were embedded in resin and their cross section was exposed by polishing, following the method described above (see §2.3). Then, the resin blocks were rinsed with Milli-Q water, air-dried and securely mounted onto the sample holder for LA-ICP-MS. Elements, including boron (^11^B), sodium (^23^Na), magnesium (^24^Mg), calcium (^43^Ca) and uranium (^238^U), were analysed. A laser source of 193 nm was used for sampling on the surface of calcified structures with a total acquisition time of 0.28 s (fluence: 3.0 J cm^–2^; spot size: 67 µm; repetition rate: 10 Hz). Before sampling, each laser spot was pre-ablated to clean the surface. Five random locations on each calcified structure were measured to obtain an average value (*n* = 5 samples per sampling area). To calibrate the data as well as correct for mass bias and instrument drift, an aluminosilicate glass reference standard (NIST612) was analysed twice at the beginning, after every 15 samples and at the end of the analysis. The recovery for the NIST 612 was 99.9% with relative standard deviation (RSD < 1.04% for all elements (*n* = 44). For quality analysis/quality control, a synthetic glass reference material (BCR-2g, United States Geological Survey) was analysed. The recovery for the BCR-2g was 102.0% with RSD < 4.8% for all elements (*n* = 22). Elemental concentrations were expressed as mmol per mol of calcium, which was chosen as the internal standard element with a value of 38.9% weight.

### (e) Statistical analyses

For the analyses with multiple measurements from the same individual, including nanoindentation, bending test and LA-ICP-MS, the values of multiple measurements (i.e. technical replicates) were averaged to reduce the impact of random error. The average value for each individual was treated as an independent sample (i.e. biological replicate) for statistical analysis. Two-way analysis of variance (ANOVA) with ‘site’ (control and vent) as the fixed factor and ‘location’ (North and South) as the random factor was applied to test their effects on all the aforementioned variables (except those expressed as percentage data) using software PRIMER 6 www.primer-e.com with PERMANOVA+ add on. The percentage data (i.e. relative weight of body components as well as porosity and organic matter content of calcified structures) were modelled using generalized linear mixed models with beta distribution. The conditional model included ‘site’ and random intercepts for ‘location’. Heterogeneity in dispersion was modelled as a function of ‘site’. The likelihood ratio test was used to assess the random effect significance. This analysis was performed using R v. 4.5.0 www.r-project.org with package glmmTMB v. 1.1.12.

## 3. Results

### (a) Body condition of sea urchins and morphology of their calcified structures

**Figure 1.**
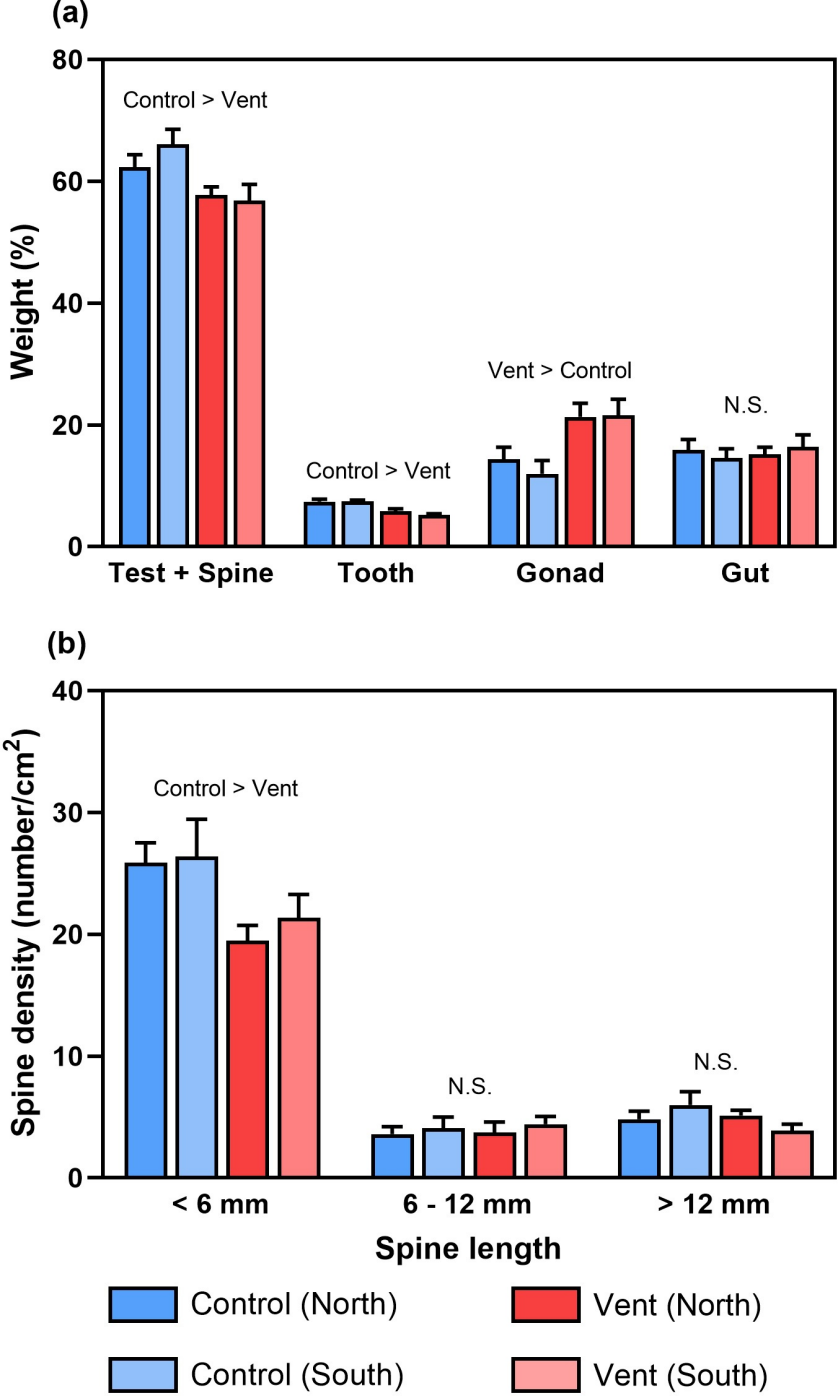
(a) The relative wet weight of tests + spines, teeth, gonads and guts of sea urchins, and (b) the density of different types of spines at different locations of the control and vent sites (mean + s.e., *n* = 5). N.S., not significant.

We found that the sea urchins at vents had lower relative weight for each calcified structure (i.e. tests, spines and teeth) than controls in both locations, by 10.8% for tests plus spines and 26.1% for teeth (figure 1a, electronic supplementary material, table S3). In contrast, the gonads of the sea urchins at vents had a higher relative weight than those at controls by 63.7%. The relative weight of guts was similar across four sampling areas. The density of primary spines (6–12 mm and >12 mm) remained similar across four sampling areas, but that of secondary spines (<6 mm) was reduced at vents by 22.0% (figure 1b, electronic supplementary material, table S4).

**Figure 2.**
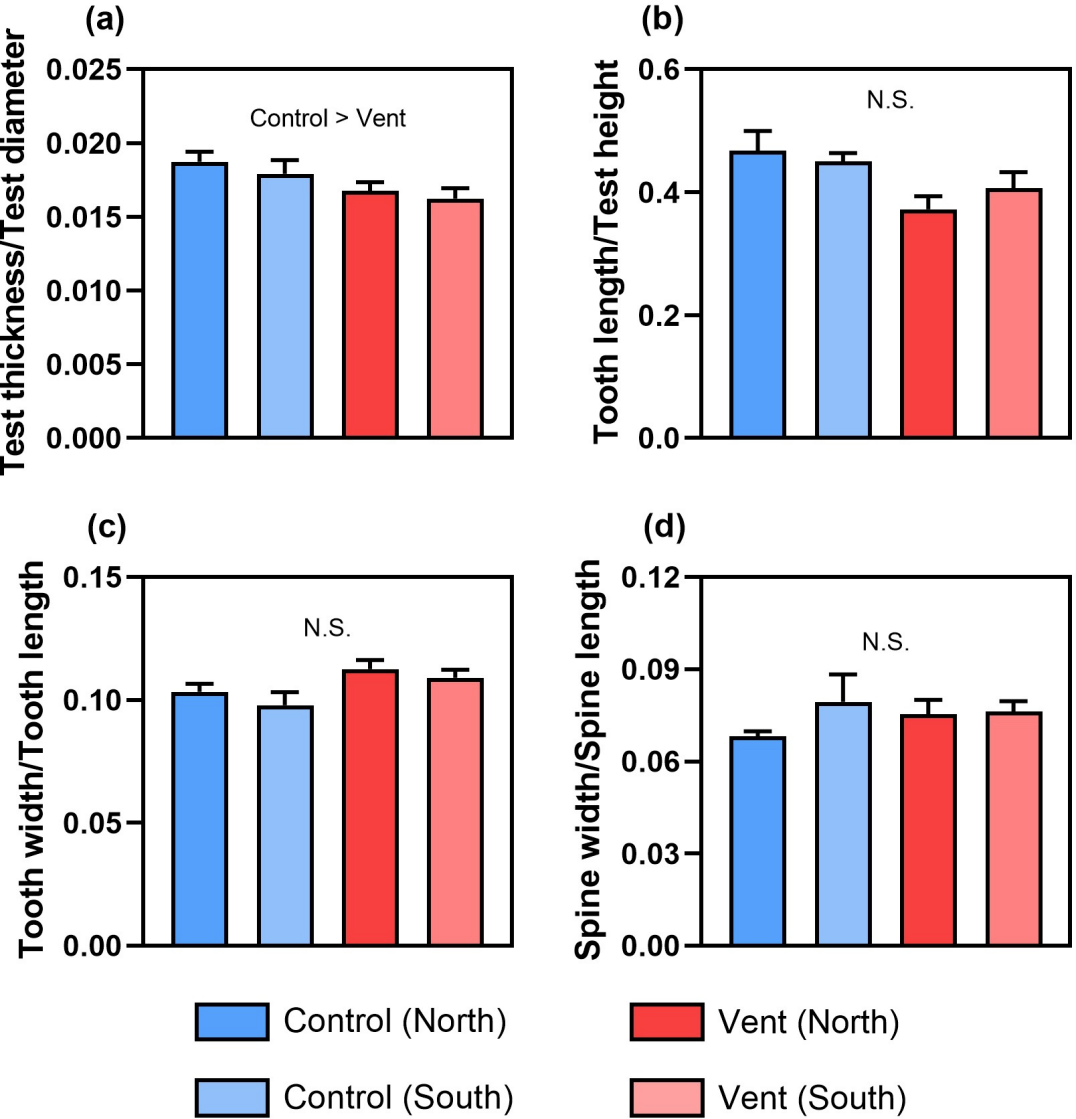
The morphology of calcified structures, including (a) test thickness/test diameter, (b) tooth length/test height, (c) tooth width/tooth length and (d) spine width/spine length of sea urchins at different locations of the control and vent sites (mean + s.e., *n* = 5). N.S., not significant.

**Figure 3.**
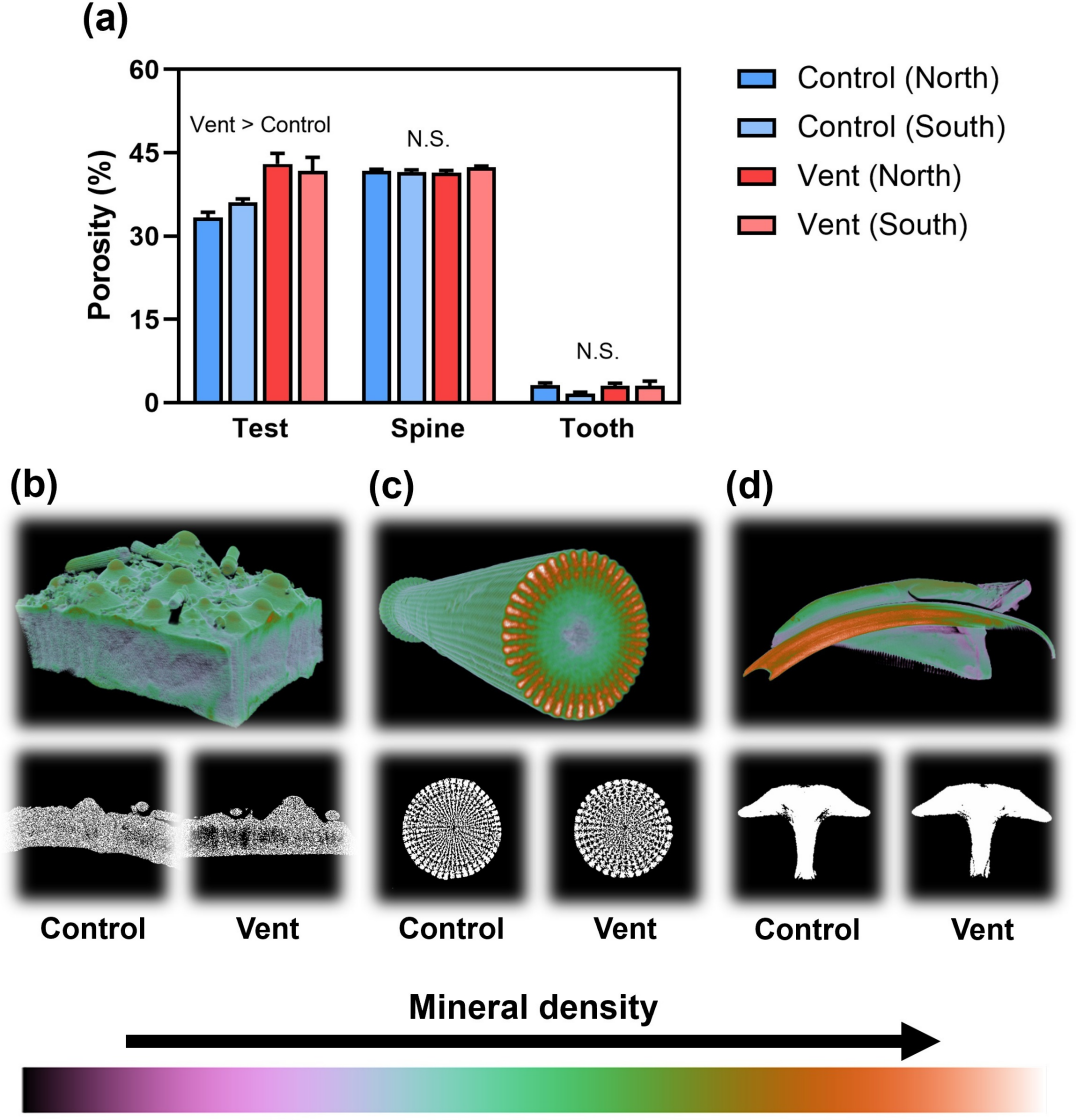
(a) The porosity of calcified structures at different locations of the control and vent sites (mean + s.e., *n* = 5). Also shown are representative three-dimensional images of sea urchin tests (b), spines (c) and teeth (d), as well as their cross-sectional images showing the porosity at the different sites. N.S., not significant.

Regarding the morphology of calcified structures, the sea urchins at vents produced thinner tests than those at controls, indicated by the lower test thickness to test diameter ratio by 9.8% (figure 2a, electronic supplementary material, table S4). The relative length of teeth at vents was slightly shorter than that at controls, indicated by the lower tooth length to test height ratio by 15.0% (figure 2b, electronic supplementary material, table S4). The teeth of sea urchins at vents appeared to be wider than those at controls, indicated by the higher tooth width to tooth length ratio by 10.4% (figure 2c, electronic supplementary material, table S4). The morphology of spines, represented by spine width to spine length ratio, remained unchanged across four sampling areas (figure 2d, electronic supplementary material, table S4). As for porosity, we found that the sea urchin tests, but not their spines and teeth, were more porous at vents than those at controls (figure 3, electronic supplementary material, table S3). Sampling location had no significant effect on the morphology of the calcified structures tested.

### (b) Mechanical properties of sea urchin calcified structures

**Figure 4.**
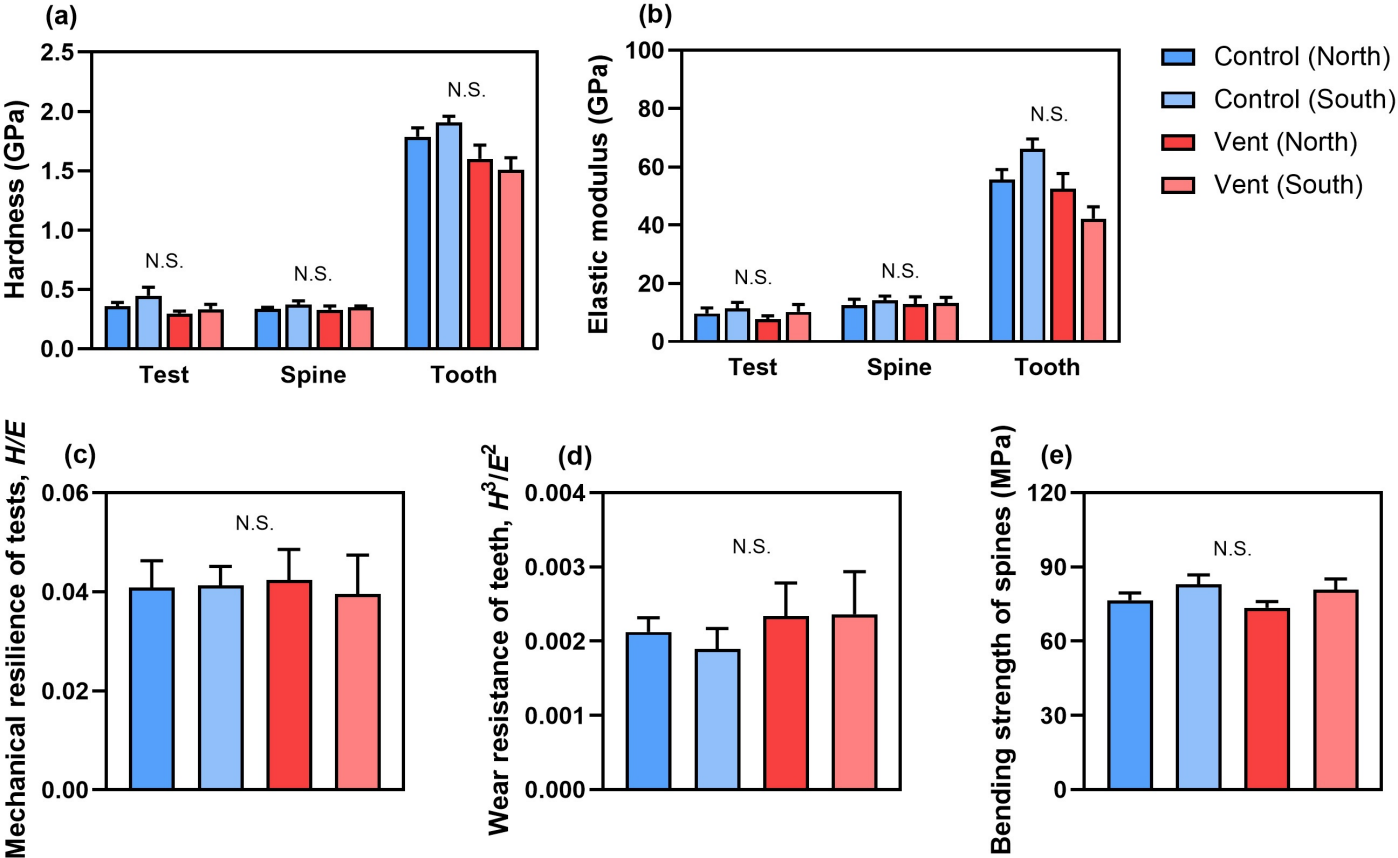
(a) The hardness and (b) elastic modulus of different calcified structures, as well as the (c) mechanical resilience of tests, (d) wear resistance of teeth and (e) bending strength of spines from sea urchins at different locations at the control and vent sites (mean + s.e., *n* = 5). N.S., not significant.

The teeth of sea urchins were harder and stiffer than their tests and spines (figure 4a,b). The teeth of sea urchins at vents appeared to have lower hardness and elastic modulus than those of urchins at controls, while the mechanical properties of tests and spines were not significantly different across four sampling areas (electronic supplementary material, table S4). The mechanical resilience of tests, wear resistance of teeth and bending strength of spines were similar between sea urchins from controls and vents (figure 4c–e, electronic supplementary material, table S4).

### (c) Chemical properties of sea urchin calcified structures

**Figure 5.**
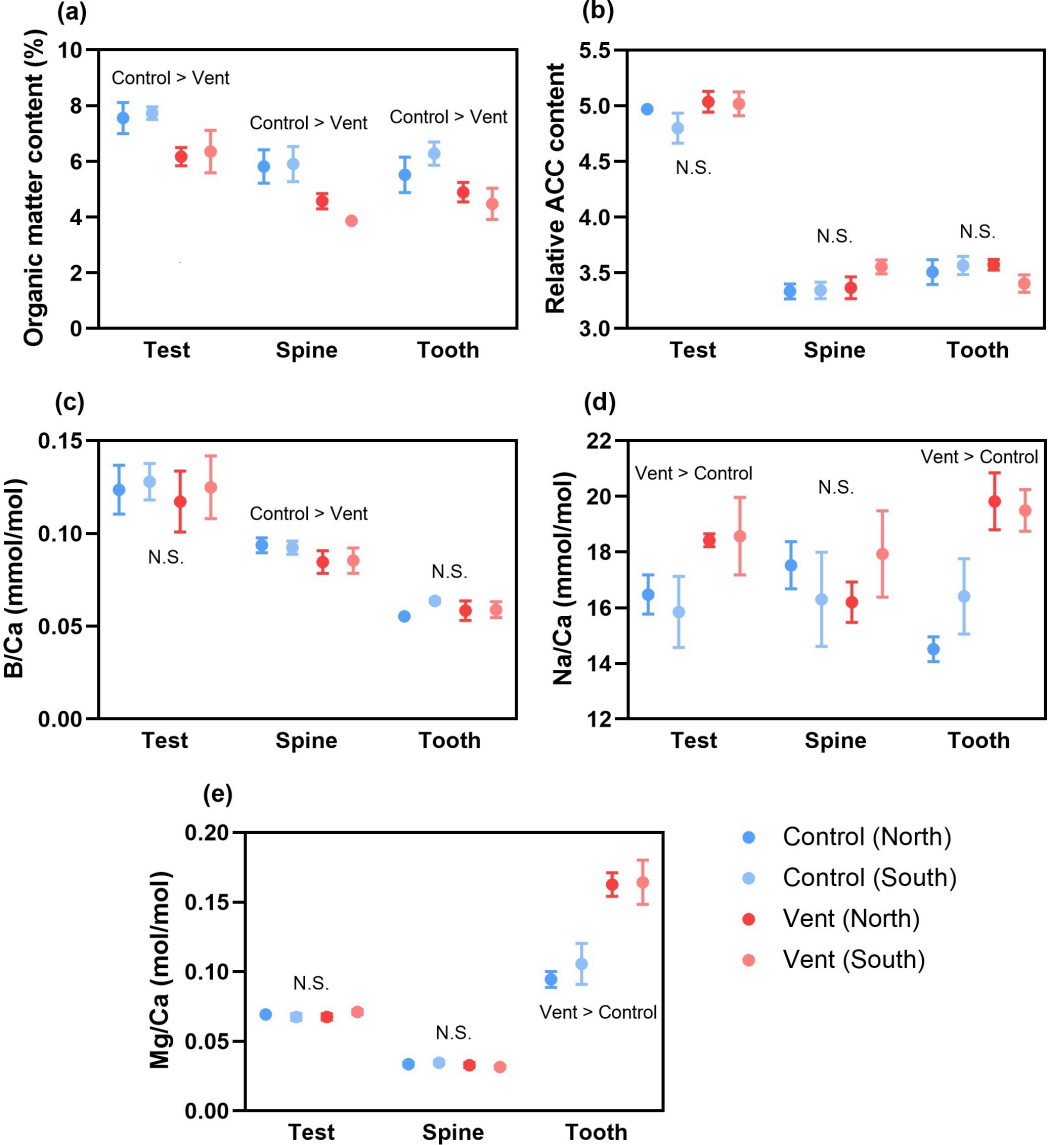
The chemical properties of calcified structures, including (a) organic matter content, (b) relative ACC content, (c) B/Ca, (d) Na/Ca and (e) Mg/Ca of sea urchins at different locations of the control and vent sites (mean ± s.e., *n* = 5). N.S., not significant.

All three calcified structures had lower organic matter content at vents than at controls, regardless of the sampling locations (figure 5a, electronic supplementary material, table S3). The spines and teeth were more crystalline (i.e. had lower relative ACC content) than tests, but the crystallinity of all three calcified structures was similar between sea urchins from controls and vents (figure 5b, electronic supplementary material, table S4). B/Ca in the tests and teeth of sea urchins was not significantly different across the four sampling areas, but slightly reduced in the spines at vents (figure 5c, electronic supplementary material, table S4). Compared with controls, Na/Ca in the tests and teeth was elevated at vents, but not in the spines (figure 5d, electronic supplementary material, table S4). The tests and spines had similar Mg/Ca across four sampling areas, but the teeth at vents had higher Mg/Ca than those at controls (figure 5e, electronic supplementary material, table S4).

## 4. Discussion

Construction of calcified structures by marine organisms can be hampered by ocean acidification, possibly reducing their fitness and survival. Using sea urchins that persist in naturally acidified habitats, we found that their calcified structures remained mechanically functional but decreased in size under ocean acidification (i.e. reduced test thickness, teeth length and spine density). These morphological changes suggest that sea urchins have the ability to prioritize the quality of calcification over its quantity, which is favourable for their survival in an acidifying ocean.

### (a) Modifications of calcified structures under ocean acidification

The test of sea urchins plays a vital role in their survival because it is the key structure for physical protection against predator attacks or hydrodynamic forces. We found more porous and thinner calcareous tests produced by sea urchins living at CO_2_ vents compared with controls, leading to reduced relative test weight. This increased test porosity, which is probably caused by impaired skeletogenesis under ocean acidification [14], may increase sea urchins’ vulnerability to predator attacks, possibly owing to reduced structural integrity and mechanical strength. For example, at a reduced pH of 7.6 the apical plates of sea urchin *Heliocidaris erythrogramma* show an increase of approximately 14% in their porosity, which is associated with their reduced hardness [39]. The increased test porosity may also be driven by the change in food quality at vents since diet can indirectly affect the formation mechanism of calcified structures [21,40]. In this study, however, the mechanical properties of tests (i.e. hardness, elastic modulus and mechanical resilience) were unaffected by ocean acidification, despite the increase in porosity by approximately 22%. This finding suggests that increased porosity does not necessarily reduce mechanical strength, and the presence of small pores may even strengthen calcified structures by facilitating energy dissipation [41]. The observed maintenance of tests’ mechanical performance may also be mediated by altering the morphology or nanostructures of calcium carbonate crystals [28,42], which warrants further investigation. Irrespective of the underlying mechanisms, our findings show that the physical protection of tests was not weakened by ocean acidification, which is fundamental to the survival of sea urchins.

Apart from the tests, spines also offer physical protection against predator attacks. While sea urchin spines can be regenerated, their formation is prone to ocean acidification [43], which may cause erosion and thus undermine their protective functions. Previous studies showed that the spines of some sea urchin species become more porous and brittle after exposure to acidified seawater for several weeks (table 1). Yet, we observed that the spines of *E. chloroticus* were not more porous at vents, suggesting their resistance to acidified seawater. Sea urchin spines are covered by a thin layer of epidermis, which can protect them from direct exposure to external seawater and hence its ‘corrosive’ effect [35,51]. As the morphology (i.e. spine width to spine length ratio) and porosity of spines were unaffected by ocean acidification, their mechanical properties and performance were maintained (table 1). Nevertheless, we found that the density of secondary spines (<6 mm) was reduced at vents, whereas the density of primary spines (>6 mm) was maintained. Although this change does not undermine the overall physical protection afforded by spines, it implies that spine quality may be maintained at the expense of spine quantity under ocean acidification.

**Table 1.** A summary of previous studies illustrating the effect of ocean acidification on the mechanical properties of calcified structures produced by different sea urchin species.

sea urchin species	pH/*p*CO_2_ level	exposure duration	effect of ocean acidification on the mechanical properties of calcified structures	reference
*Strongylocentrotus droebachiensis*	47, 102 and 284 Pa	6 weeks	Breaking force of spines unchanged at 102 Pa, but reduced at 284 Pa	[44]
*Paracentrotus lividus*	390, 550, 750 and 1000 μatm	1 month	Test robustness unchanged at 550 and 750 μatm, but reduced at 1000 μatm	[45]
*Tripneustes gratilla*	pH 8.1, 7.8 and 7.6	146 days	Crushing force of tests unchanged at pH 7.8, but reduced at 7.6	[46]
*Echinometra mathaei*	pH 8.08 and 7.63	12 months	For both ambital and apical plates, force at rupture, flexural stiffness and Young’s modulus unchanged	[47]
*Paracentrotus lividus*	380, 750 and 1000 μatm	49 weeks	For both ambital and apical plates, hardness, force at fracture and Young’s modulus unchanged	[34]
*Paracentrotus lividus*	pH 8.21 and 7.78	N/A (from the CO_2_ seep sites)	For both ambital and apical plates, hardness, force at fracture and Young’s modulus unchanged	[34]
*Lytechinus variegatus*	pH 7.93, 7.70 and 7.47	59 days	Spine load-bearing weight unchanged at pH 7.70, but reduced at pH 7.47	[48]
*Loxechinus albus*	400 and 1200 μatm	7 months	Force required to cause the failure of structural integrity unchanged	[36]
*Eucidaris tribuloides*	pH 8.1, 7.7 and 7.4	5 weeks	Fracture force and Young’s modulus of both primary and secondary spines unchanged	[35]
*Tripneustes ventricosus*	pH 8.1, 7.7 and 7.4	5 weeks	Fracture force of primary and secondary spines unchanged at pH 7.7, but reduced at 7.4; Young’s modulus of both primary and secondary spines unchanged	[35]
*Paracentrotus lividus*	pH 8.1, 8, 7.8 and 7.7	1 month	Fracture force and Young’s modulus of both ambital and apical plates unchanged	[49]
*Paracentrotus lividus*	pH 7.93 and 7.63	N/A (from the CO_2_ vents)	Force at fracture, stress and elastic modulus of both ambital and apical plates unchanged	[50]
*Arbacia lixula*	pH 7.93 and 7.63	N/A (from the CO_2_ vents)	Force at fracture, stress and elastic modulus of both ambital and apical plates reduced	[50]
*Heliocidaris erythrogramma*	pH 8.01 and 7.60	9 months	Hardness and Young’s modulus of apical plate reduced	[39]
*Paracentrotus lividus*	pH 8.04 and 7.78	3 months	Test robustness unchanged	[43]
*Sterechinus neumayeri*	pH 8.13, 8.04, 7.77 and 7.83	N/A (from the CO_2_ vents)	Elastic modulus and stress of interambulacral ambital plates unchanged; fracture force of interambulacral ambital plates reduced at one of the acidified sites	[37]

The feeding organ of sea urchins, Aristotle’s lantern, comprises five enclosed teeth. Apart from grazing benthic algae for food acquisition, the lantern is also responsible for locomotion, burrowing and rock-boring. Thus, sea urchin teeth are much more mineralized (porosity <5%) than tests and spines, which accounts for their higher hardness and stiffness. How ocean acidification affects sea urchin teeth is less studied than tests and spines, but acidified seawater has been shown to disrupt the growth of sea urchin teeth in terms of size and shape [52]. Here, we found that the teeth of sea urchins living at vents appeared to be slightly softer but less stiff so that the wear resistance of teeth was maintained (cf. sea urchins from controls). In addition, the sea urchin teeth at vents tended to be wider (i.e. increased tooth width to tooth length ratio) and shorter (i.e. reduced tooth length to test height ratio), which can increase their resistance to fracture [53]. However, the shorter teeth indicate reduced structural size under ocean acidification, possibly accounting for the reduced relative weight of teeth. Since the mechanical performance of teeth remained unchanged, this morphological change may not be unfavourable because more energy can be diverted from calcification to other physiological processes, such as growth of non-calcified structures [9]. This may account for the higher relative gonad weight of some sea urchin species at vents than controls [54], but the mechanism underlying this change requires further investigation with more individuals tested (*n* > 10 per treatment group) to have a more statistically robust interpretation.

Apart from morphology and porosity, Mg content may also influence the mechanical properties of calcified structures. Previous studies showed that increased Mg content can boost mechanical strength and impede crack propagation in calcified structures [55]. This notion can explain the higher hardness and stiffness of sea urchin teeth than tests and spines [22], which is biologically important as the teeth experience greater loads than tests and spines for grazing hard surfaces. Here, we observed that the Mg content of sea urchin teeth, but not of tests and spines, was higher under elevated CO_2_ conditions (see also ref. [46]). The underlying mechanism of increased Mg incorporation in teeth under elevated CO_2_ conditions remains enigmatic but may be related to the change in organic components that affect crystal growth and Mg incorporation into the calcite lattice [56]. Regardless of the underlying mechanisms, the increase in the Mg content of teeth did not alter their mechanical properties, suggesting that the hardening effect of Mg on calcified structures has a limit. This proposition is substantiated by an observation that the hardness of calcium carbonate minerals is associated not with their Mg content, but with their crystallinity or crystal domain volumes [42]. Other factors may also determine the mechanical strength of calcified structures, such as the presence of specific macromolecules and crystal orientation [55], which deserves further investigation. Overall, our integrated analysis using different techniques helps reveal how changes in morphological and structural properties can influence the functionality of calcified structures under ocean acidification.

### (b) Trade-offs of morphological plasticity in sea urchins

Ocean acidification is expected to disrupt calcification, resulting in the production of less functional calcified structures (e.g. mechanically weaker shells or skeletons [57]). For example, gastropods (*Charonia lampas*) living near CO_2_ seeps have thinner, more porous shells owing to shell erosion by acidified seawater [58]; corals (*Stylophora pistillata*) have stunted aragonite growth under ocean acidification owing to reduced pH of extracellular fluid at the tissue–skeleton interface [59]. Contrary to this prediction, the sea urchins were found to maintain crystallization of calcium carbonate in all three types of calcified structures under ocean acidification (i.e. unaltered relative ACC content), suggesting that the quality of calcium carbonate crystals was not reduced [17,18,60]. This observation can be mediated by the capacity of sea urchins to regulate the acid–base balance of calcifying fluid (e.g. through accumulation of bicarbonate ions or activation of Na^+^/H^+^ exchanger [61,62]), whereby an optimal alkaline condition at the calcification site can be sustained. Here, the increased Na/Ca in the calcified structures suggests that the sea urchins were able to activate the Na^+^/H^+^ exchanger to remove excessive protons under ocean acidification so that the carbonate saturation of calcifying fluid was maintained (i.e. unaltered B/Ca) for precipitation of calcium carbonate crystals. Indeed, the capacity to regulate acid–base balance can determine the quality of calcified structures produced as well as the sensitivity of sea urchins to ocean acidification. For example, sea urchin *Paracentrotus lividus* has a higher capacity to adjust the acid–base balance of extracellular fluid than *Arbacia lixula*; therefore, the former is able to maintain the mechanical strength of calcified structures produced under ocean acidification [50].

Although acid–base regulation can help maintain the quality of calcified structures produced under ocean acidification, this compensatory mechanism is fuelled by energy and hence incurs energy trade-offs [2,63]. In this regard, less energy can be allocated to the production of calcified structures, probably accounting for the reduced relative size and weight of tests, spines and teeth. Indeed, the capacity of marine calcifiers to construct their calcified structures decreases with decreasing availability of metabolic energy produced chiefly by aerobic metabolism, and *vice versa* [64,65]. While size reduction in calcified structures is generally considered unfavourable, it can be beneficial by allowing reallocation of energy to other physiological processes to adjust to the changing environmental conditions [7,66]. For instance, brachiopods and foraminifera can simplify their morphological features (e.g. reduced number of shell plicae, shell thickness and shell ornamentation) as an energy-saving strategy to cope with mass extinction events [9]. Similarly, bryozoans (*Myriapora truncata*) can build thicker skeletons to counter acidified conditions via energy reallocation to calcification, but this strategy leads to reduced zooid volume as energy trade-offs against overall growth [67]. As long as the functionality of calcified structures is not undermined, this strategy can favour the survival of marine calcifiers in an acidifying ocean, like the sea urchins in this study. Indeed, reduced structural size may serve as an evolutionary strategy of marine organisms in response to climate change [32,68].

### (c) Implications for the calcifying capacity of sea urchins in an acidifying ocean

Based on the observations in many earlier studies, there is concern that sea urchins may lose their ability to construct calcified structures in the future owing to their high sensitivity to ocean acidification. Yet, this proposition is derived largely from short-term experiments (e.g. [61,69]), which largely ignored natural ecological complexity or the acclimation capacity of sea urchins. We now increasingly recognize that sea urchins can maintain the integrity of their calcified structures under ocean acidification when long exposure time (more than 6 months) is given for acclimation. For instance, compared with the control at pH 8.1, no sign of erosion on calcified structures is observed in sea urchin *Echinometra* sp. when exposed to ocean acidification at pH 7.7 for 11 months [51]; the mechanical properties (e.g. nanohardness, Young’s modulus, fracture force, etc.) of the skeleton of sea urchin *P. lividus* and *Echinometra mathaei* are maintained at a natural CO_2_ vent after 6 month exposure, indicating the maintenance of test integrity [34,47]. Thus, we suggest that some sea urchin species can perform and survive well in an acidifying ocean via long-term acclimation.

Apart from short-term experimental exposure, the use of highly acidified seawater for experimentation can also increase the likelihood of obtaining negative effects (table 1). For example, the growth of sea urchin *Tripneustes gratilla* can be sustained at pH 7.8, but not at pH 7.6 [70], suggesting that the ability of sea urchins to adjust to reduced seawater pH has a limit beyond which their fitness is reduced. Since the commonly used business-as-usual scenario of CO_2_ emissions is considered implausible in the future [71], it is necessary to rethink the pessimism about the future survival of sea urchins [3,72]. Whether sea urchins resist or succumb to ocean acidification would be largely determined by their ability to maintain energy homeostasis, which can support various compensatory mechanisms, including acid–base regulation [2]. Since such ability is species-specific [73], more species should be examined in the future using more sophisticated experimental design to generalize whether sea urchins can adapt to ocean acidification [3]. Furthermore, more studies on sea urchin larvae using ecologically relevant settings are needed because larvae are considered more sensitive than adults to ocean acidification and may be the bottleneck of population persistence [2,23,74,75].

## 5. Conclusion

**Figure 6.**
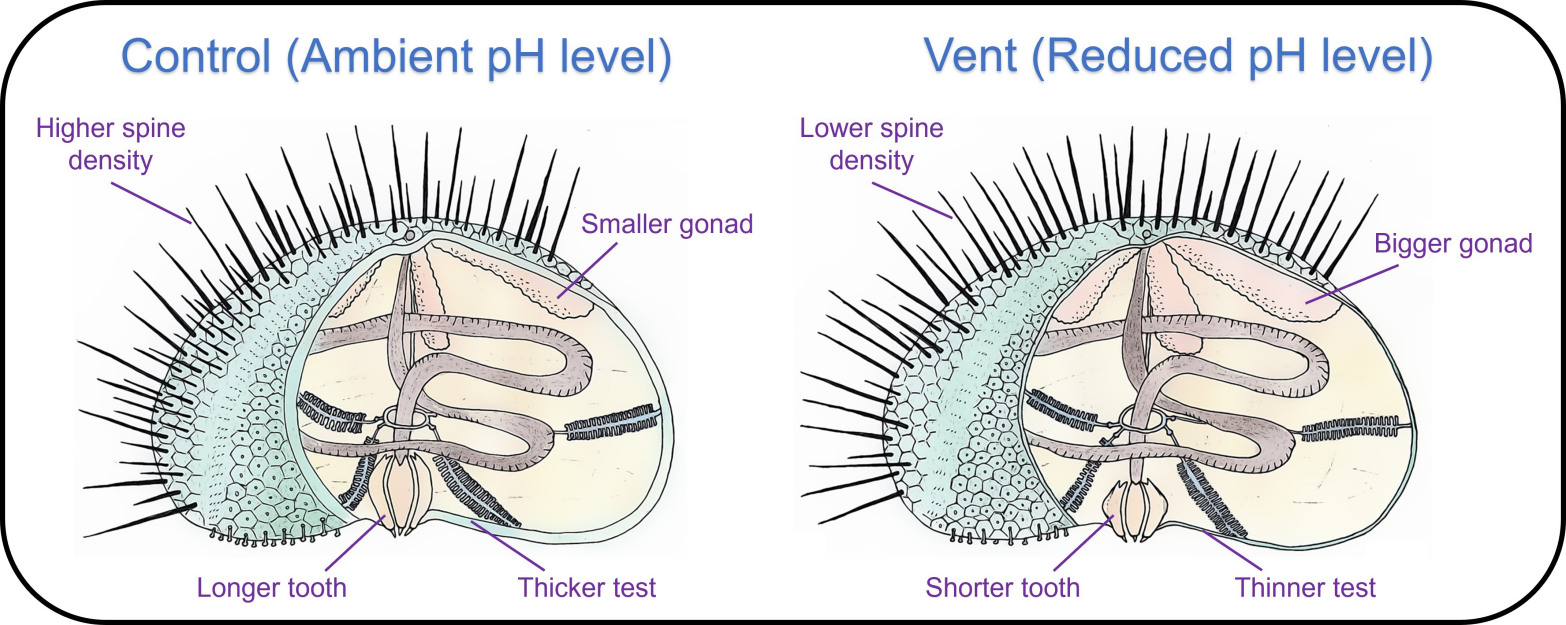
A conceptual diagram showing the differences in the morphology of calcified structures and gonad size of sea urchins between the control and vent sites. For sea urchins inhabiting natural CO_2_ vents, morphological shifts in calcified structures were observed (i.e. thinner tests, shorter teeth and fewer spines), while gonad size was enlarged (compared with controls).

To adjust to the long-term increase in seawater acidity, morphological shifts in calcified structures may act as a plastic, intragenerational response, but how this is linked to structural performance has been underappreciated. Here, we unveiled that the calcified structures of a sea urchin species remained fully functional under ocean acidification, despite their reduction in size (i.e. thinner tests, smaller teeth and lower spine density, figure 6). Nevertheless, this strategy is subject to the energy balance of sea urchins, which may decrease under ocean acidification [70]. As anthropogenic CO_2_ emissions in the future are likely to be more strictly regulated [71], ocean acidification would be slower and less severe, and its impacts on marine organisms would be weaker than previously thought. Our new understanding of the interplay between morphological, structural and chemical properties of calcified structures to maintain their performance suggests that we should reconsider the vulnerability of sea urchins to ocean acidification, as they may have sufficient time to acclimate or even adapt through morphological shifts and other compensatory mechanisms (e.g. mineralogical adjustment, energy reallocation and acid–base regulation [2,76]) in response to ocean acidification. Therefore, if the adaptive capacity of sea urchins could keep pace with the rapid changes in seawater chemistry under ocean acidification [77], they could prevail and maintain their ecological roles in a future high-CO_2_ world.

## Data Availability

Data of this work are available online in electronic supplementary material [78].
